# Clinicoradiological outcomes after radical radiotherapy for lung cancer in patients with interstitial lung disease

**DOI:** 10.1259/bjro.20220049

**Published:** 2023-04-19

**Authors:** Gerard M Walls, Michael McMahon, Natasha Moore, Patrick Nicol, Gemma Bradley, Glenn Whitten, Linda Young, Jolyne M O'Hare, John Lindsay, Ryan Connolly, Dermot Linden, Peter A Ball, Gerard G Hanna, Jonathan McAleese

**Affiliations:** 1 Cancer Centre Belfast City Hospital, Belfast Health & Social Care Trust, Lisburn Road, Belfast, Northern Ireland; 2 Patrick G Johnston Centre for Cancer Research, Queen’s University Belfast, Belfast, Northern Ireland; 3 Department of Respiratory Medicine, Belfast Health & Social Care Trust, Belfast, Northern Ireland; 4 Department of Radiology, Belfast Health & Social Care Trust, Belfast, Northern Ireland

## Abstract

**Objective:**

Interstitial lung disease (ILD) is relatively common in patients with lung cancer with an incidence of 7.5%. Historically pre-existing ILD was a contraindication to radical radiotherapy owing to increased radiation pneumonitis rates, worsened fibrosis and poorer survival compared with non-ILD cohorts. Herein, the clinical and radiological toxicity outcomes of a contemporaneous cohort are described.

**Methods:**

Patients with ILD treated with radical radiotherapy for lung cancer at a regional cancer centre were collected prospectively. Radiotherapy planning, tumour characteristics, and pre- and post-treatment functional and radiological parameters were recorded. Cross-sectional images were independently assessed by two Consultant Thoracic Radiologists.

**Results:**

Twenty-seven patients with co-existing ILD received radical radiotherapy from February 2009 to April 2019, with predominance of usual interstitial pneumonia subtype (52%). According to ILD-GAP scores, most patients were Stage I. After radiotherapy, localised (41%) or extensive (41%) progressive interstitial changes were noted for most patients yet dyspnoea scores (*n* = 15 available) and spirometry (*n* = 10 available) were stable. One-third of patients with ILD went on to receive long-term oxygen therapy, which was significantly more than the non-ILD cohort. Median survival trended towards being worse compared with non-ILD cases (17.8 *vs* 24.0 months, *p* = 0.834).

**Conclusion:**

Radiological progression of ILD and reduced survival were observed post-radiotherapy in this small cohort receiving lung cancer radiotherapy, although a matched functional decline was frequently absent. Although there is an excess of early deaths, long-term disease control is achievable.

**Advances in knowledge:**

For selected patients with ILD, long-term lung cancer control without severely impacting respiratory function may be possible with radical radiotherapy, albeit with a slightly higher risk of death.

## Introduction

Interstitial lung disease (ILD) is relatively common in patients with primary lung cancer, with an incidence of 7.5% cases.^1^ Lung cancer shares common risk factors with ILD, such as smoking and chemical exposure, which may partially account for their co-occurrence.^2,3^ ILD has been shown to be a risk factor for the development of lung cancer, independent of smoking status.^4^ The relative risk of lung cancer is 3.5–7.3 times higher in ILD, with over 15% patients thought to die from lung cancer.^2,5^

Cases of lung cancer unsuitable for curative surgery due to technical factors or co-morbidity may be approached radically with radiotherapy (RT). In terms of local control, RT is comparable with surgery for smaller tumours [stereotactic ablative RT (SABR)], but for larger tumours, RT is inferior to surgery (conventional RT).^6^ The radiation dose deliverable to lung tumours is restricted by the tolerance of adjacent normal tissues, predominantly the pulmonary parenchyma, cardiac structures and spinal cord.^7^ The characteristic pulmonary reserve-depleted phenotype of ILD demands special consideration during RT planning due to the narrowed therapeutic index.^8–11^

Historically RT was contraindicated in significant ILD due to increased rates of radiation pneumonitis (RP) at 24–43%.^2,12^ Subclinical ILD is also associated with increased ≥Grade 2 RP, with 36% patients with ILD developing RP compared with 13% patients without.^4^ Irradiation of more than 10% of the lung field has been shown to be an independent risk factor of RP.^13^ Pre-treatment ILD and mean lung dose are also associated with RP in SABR cohorts.^14^

Survival outcomes have also been shown to be negatively impacted by the presence of ILD for conventional RT.^15,16^ A study of non-small cell lung cancer (NSCLC) with co-existing ILD treated with SABR, with a median 3-year overall survival (OS) of 48% compared to 68% for non-ILD patients.^8^ Fatal respiratory complications have been reported following SABR in cases of subclinical ILD also.^2,10,11^

In this study, the pulmonary impact of radical RT in the setting of ILD is evaluated by means of functional and radiological parameters in a cohort of prospectively evaluated patients, and both supplementary oxygen requirement and OS are compared with a large non-ILD cohort treated at the same centre in the same time period.

## Patient and materials

### Study population

Patient details were prospectively collected following discussion at a Consultant-led regional RT peer review meeting between 2009 and 2019. The diagnosis and characterisation of ILD was jointly made by the Consultant Radiologist and Respiratory Physician reviewing new diagnoses of lung cancer at a previous regional thoracic oncology multidisciplinary meeting (MDM). Details of non-ILD cases of lung cancer discussed at the same MDM and peer review meeting were also recorded for comparison.

Case records were interrogated for baseline patient characteristics including smoking status, Charlson Comorbidity Index (CCI) and World Health Organisation Performance Status (WHO-PS). A staging^18^FDG-PET/CT was mandatory for staging during this period and cross-sectional brain imaging was optional. The regional protocol for routine follow-up consists of 3-monthly clinical assessments and CT chest/abdomen at 3 months and 2 years post-RT for conventional treatments, but 6-monthly for the first year and annually thereafter following SABR. From years 3 to 5, patients are followed clinically with 6-monthly reviews.

### Baseline pulmonary assessments

Baseline ILD was characterisedby MRC Dyspnoea Scores (MRCDS), a semi-quantitative clinician-assessed grade of breathlessness^17^ and by ILD-GAP scores, retrospectively generated from baseline patient demographics, pre-treatment spirometry parameters and ILD subtype (according to Ryerson et al^18^). Baseline ILD was characterised radiologically by semi-quantitative visual assessment of the mediastinal and lung windows of RT planning CT scans by two Consultant Thoracic Radiologists according to accepted criteria.^19^ Radiological ILD subtypes present and their severity were recorded based on semantic imaging features.^20^

### Radiotherapy

AllRT was planned with three-dimensional conformal RT (3DRT), or intensity modulated RT (IMRT) techniques including volumetric modulated arc therapy (VMAT). Cases treated after 2012 had internal target volumes (ITVs) created on four-dimensional CT (4DCT) planning scans to compensate for respiratory motion. Prior to this, clinical target volumes were generated with population-based margins on three-dimensional scans. Image-guidance was largely achieved with cone-beam CT scanning in the study period. The dose and fractionations used to treat NSCLC were 55 Gy/20# over 4 weeks or 60–66 Gy/30–33# over six and a half weeks. Patients with SCLC were treated with either 40 Gy/15# over 3 weeks, 45 Gy/30# over 3 weeks or 50 Gy/25# over 5 weeks. SABR cases received 54 Gy/3# or 55–60 Gy/5–8#. All target volumes were subject to peer review.^21^ Cases were selected for concurrent or sequential chemotherapy based on WHO-PS and co-morbidity burden. Patients were routinely clinically assessed once per week by a Clinical Oncologist or Specialist Therapeutic Radiographer. Planning parameters recorded for this study were planning target volume (PTV) size, algorithm used, volume of lung receiving 5 Gy (V5), volume of lung receiving 18 or 20 Gy (V18/20) and mean lung dose (MLD).

### Re-assessment of pulmonary disease

The occurrence, grade [Common Terminology Criteria for Adverse Events (CTCAE)^22^] and steroid treatment of radiation pneumonitis were recorded on clinical follow-up. RT response assessment scans at 2 years were assessed by two Consultant Thoracic Radiologists. Where a ‘2 year’ scan was not available, the most recent scan was used. Grading was applied to the interstitial appearances^19^ and note was made of whether progression was localised to the RT field region or extended beyond this. The most recent MRCDS grading was recorded from the medical notes where available. Repeat spirometry where available was compared with baseline in terms of clinical significance, defined as an absolute decrease in predicted forced vital capacity (FVC) of ≥10 percentage points**,** for transfer factor, of ≥15 percentage points, and for forced expiratory in 1 second (FEV1) by ≥12 percentage points.^23,24^ Long-term oxygen therapy (LTOT)-free survival was calculated, defined as the interval between starting radiotherapy and the commencement of LTOT in patients needing supplemental oxygen.

### Overall survival

Survival was taken from the start date of RT until death or last known follow-up. Follow-up data of consecutive patients without ILD treated in the same time period were used for comparison of overall and lung cancer-specific survival, as well as LTOT-free survival.

### Statistics

Descriptive statistics were used to summarise baseline patient and tumour characteristics. Paired two-sided *t* tests were used to test differences between baseline and post-treatment pulmonary function tests. Multivariate analysis was performed to assess the association of pre-specified clinical factors with the onset of LTOT requirement. Kaplan–Meier survival analysis and log rank tests were used to compare survival between the ILD cohort and all other patients receiving radical RT in the same time frame.

## Results

### Patient and tumour characteristics

Twenty-seven patients with ILD were treated with radical RT between 2009 and 2019 with a median follow-up of 1.4 years. The mean age was 73 (range 56–85), there were more male patients (56%), and most patients smoked previously (67%). The median CCI was 4 (range 1–8) and the majority of patients were WHO-PS 0–1 (63%). Most patients had confirmed adenocarcinoma (30%) or squamous cell carcinoma (30%), and both early and locally advanced disease were represented. Most patients were treated with conventional RT alone (78%), and only two patients were prescribed chemotherapy as part of their treatment, both receiving this prior to commencing RT. Apart from the latter, the patient characteristics were broadly comparable with the non-ILD cohort from the same period, as shown in Table 1.

**Table 1.** Baseline patient, tumour and treatment details

		Patients with	Patients without
		ILD (%)	ILD (%)
		*n* = 27	*n* = 1304
General	Male	15 (56)	681(52)
	Mean age (range)	73 years (56–85)	70 years (32–92)
Smoking Status	Current	8 (30)	351 (27)
	Previous	18 (67)	542(42)
	Never	1 (4)	32 (2)
	Unknown	0 (0)	379 (29)
Performance status	0	3 (11)	146 (11)
	1	14 (52)	574 (44)
	2	9 (33)	487(37)
	3	1 (4)	71 (5)
	Unknown	0	26 (2)
AJCC staging (v6-7)	T0	0	21 (2)
	T1	11 (41)	475 (36)
	T2	9 (33)	360 (28)
	T3	2 (7)	208 (16)
	T4	5 (19)	208 (16)
	N0	18 (67)	626 (48)
	N1	4 (15)	155 (12)
	N2	5 (19)	428 (33)
	N3	0	80 (6)
	Unknown	0	15 (1)
Histology	Small cell	2 (7)	211 (16)
	Squamous cell	8 (30)	421 (32)
	Adenocarcinoma	8 (30)	323 (25)
	NSCLC (Other)	0 (0)	75 (6)
	Clinical diagnosis	9 (33)	275 (21)
Treatment Paradigm	SCLC CCRT	0	65 (5)
	SCLC SCRT	1 (4)	135 (10)
	SCLC RT	1 (4)	10 (1)
	SCLC SABR	0	1 (0)
	NSCLC CCRT	0	90 (7)
	NSCLC SCRT	1 (4)	194 (15)
	NSCLC RT	21 (78)	518 (40)
	NSCLC SABR	3 (11)	291 (22)

ILD, interstitial lung disease;NSCLC, non small cell lung cancer; RT, radiotherapy; SABR, stereotactic ablative radiotherapy.

### Radiotherapy details

The moderately hypofractionated regime of 55 Gy in 20 fractions was used most commonly (67%). The mean PTV volume was 198 cc (range 17–409). Half of RT plans (52%) were created using IMRT techniques, and four of these cases were SABR plans. A majority of patients underwent a 4DCT scan for treatment planning. The median MLD and V18/20 were 8.9 Gy (range 2.9–17.8), and 14% (range 2–30), which were similar in the non-ILD cohort [median MLD 9.3 Gy (range 1–20), median V18/20 17% (range 0–38).] The V5 was 42% (range 12–78), however, this was not available for the non-ILD cohort. Kilovoltage imaging with cone-beam CT was available for most patients (93%). These radiotherapy characteristics were broadly comparable with the non-ILD cohort from the same period, as shown in Table 2.

**Table 2.** Radiotherapy planning characteristics

		Patients with	Patients without
		ILD (%)	ILD (%)
		*n* = 27	*n* = 1304
	Lung V18/20	14% (range 2–30)	17% (range 0–38)
	MLD	8.9 Gy (range 2.9–17.8)	9.5 Gy (range 1–20)
Localisation	4DCT	23 (85)	933 (71)
	3DCT	4 (15)	371 (28)
Planning	3DRT	13 (48)	743 (57)
	IMRT	1 (4)	22 (2)
	VMAT	13 (48)	539 (41)
Dose Fractionation	40 Gy / 15#	1 (4)	102 (8)
	50 Gy / 25#	1 (4)	41 (2)
	45 Gy / 30#	0	59 (4)
	55 Gy / 20#	18 (67)	708 (54)
	60–66 Gy / 30–33#	3 (11)	100 (8)
	72–79 Gy / 40–44#	0	5 (0)
	54 Gy / 3#	1 (4)	33 (3)
	55–60 Gy / 5#	3 (11)	174 (13)
	55–60 Gy / 8–10#	0	82 (6)
Image guidance	CBCT	25 (93)	904 (69)
	MV	2 (7)	400 (31)

CBCT, cone beam CT; 3DCT, three-dimensional CT; 4DCT, four-dimensional CT; 3DRT, three-dimensional radiotherapy; ILD, interstitial lung disease; IMRT, intensity modulated radiotherapy; VMAT, volumetric modulated arc therapy.

### Baseline pulmonary function

The pre-treatment MRCDS was one or two for most patients (81%). At baseline, ILD was radiologically mild (74%) or moderate (26%) in all cases and there was a predominance (52% cases) of usual interstitial pneumonia (Table 3), including five cases without honeycombing. Reticulation was the most commonly observed pattern radiologically (81%) followed by traction bronchiectasis (44%), honeycombing (41%) and ground-glass (37%). By ILD-GAP scoring, 89% patients were Stage I at baseline (*i.e.* score of ≤3).

**Table 3.** Baseline and post-treatment pulmonary parameters

		Patients with ILD (%)
		*n* = 27
Radiological Patterns	Reticulation	22 (81)
	Traction bronchiectasis	12 (44)
	Honeycombing	11 (41)
	Ground glass	10 (37)
Radiological ILD severity	Mild	20 (74)
	Moderate	7 (26)
	Severe	0 (0)
Clinical ILD Subtype	UIP	14 (52)
	Unclassifiable	8 (30)
	CT-ILD/NSIP	5
	Chronic HP	0 (0)
ILD-GAP Score	Stage I (≤3)	24
	Stage II (4–5)	3
	Stage III (6–8)	0
MRCDS	1	10 (37)
	2	12 (44)
	3	3 (11)
	4	2 (7)
Radiation Pneumonitis Grade	0	2
	1	16
	2	4
	3	5
	4	0
Post-treatment Radiological ILD progression (*n* = 27 at 11 month interval, median)	None	5 (19)
	Local	11 (41)
	Extensive	11 (41)
Post-treatment MRCDS (*n* = 15 at 12 month interval, median)	Deterioration ≥ 1 Point	6 (40)
	No change	9 (60)

ILD, interstitial lung disease; MRCDS, MRC Dyspnoea Score.

### Pulmonary toxicity

Less than a fifth of patients (*n* = 5) patients developed Grade 3 RP and one patient developed bilateral RP. Notably, all of these patients went on to require long-term oxygen therapy. After radiotherapy, almost all cases demonstrated progressive ILD changes (82%), with 41% cases demonstrating radiological progression of interstitial changes outside the treatment field. Less than half of the patients (40%) exhibited an increased MRCDS compared with baseline following radical RT at a median interval of 12 months.

Interval spirometry was available for less than half of the cohort, and differences observed were not statistically significant (Table 4) after a median interval of 11 months. Clinically significant declines in FVC, TF and FEV1 were seen in the minority. FVC declined by ≥10% in 3 of the 7 patients where it was available, transfer factor by ≥15% in 2 of 8 patients, and FEV1 by ≥12% in 3 of 10 patients. Nine (33%) patients commenced oxygen during follow-up, with a mean time to commencing oxygen of 15 months. No patients received antifibrotic treatment.

**Table 4.** Baseline and post-treatment spirometry

	Baseline (*n* = 27)		Post-treatment(*n* = 11)		*t* test
	Median (range)	n available	Median (range)	n available	
**Predicted FVC**	103 (66–135)	24	87 (64–128)	9	*p* = 0.505
**Predicted FEV1**	96 (34–149)	27	87 (65–162)	11	*p* = 0.140
**Predicted TF**	67 (35–101)	27	62 (54–87)	8	*p* = 0.385

FEV1, forced expiratory in 1 s; FVC, forced vital capacity.

**Figure 1.**
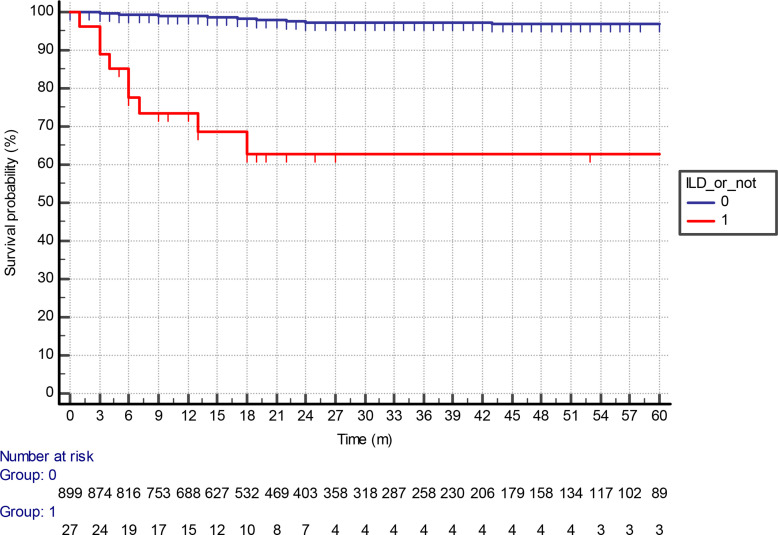
A Kaplan–Meier analysis of long-term oxygen therapy-free survival (months) for patients receiving radical radiotherapy stratified by presence or absence of interstitial lung disease.

LTOT-free survival was significantly higher for patients without ILD compared to those (*p* < 0.0001), as shown in Figure 1, although data were available for 899 patients in the ILD cohort. On multivariate analysis, only FEV1 and ILD status were significantly associated with requiring LTOT (Table 5).

**Table 5.** Multivariate analysis of clinically relevant factors for the development of a requirement for oxygen therapy

Covariate	b	SE	Wald	*p*	Exp(b)	95% CI of Exp(b)
**Pathology**	−0.4272	0.7183	0.3537	0.552	0.6523	0.1596–2.66662
**EQD2**	−0.01837	0.02064	0.7921	0.3735	0.9818	0.9429 to 1.0223
**ILD history**	3.1448	0.4928	40.7215	<0.0001	23.2153	8.8366 to 60.9910
**FEV1**	−0.02099	0.00977	4.6165	0.0317	0.9792	0.9607 to 0.9982
**TF**	−0.00403	0.01009	0.1594	0.6897	0.996	0.9765 to 1.0159
**Lung V20**	0.04087	0.02631	2.4123	0.1204	1.0417	0.9894 to 1.0969

FEV1, forced expiratory in 1 s; ILD, interstitial lung disease.

### Survival

**Figure 2.**
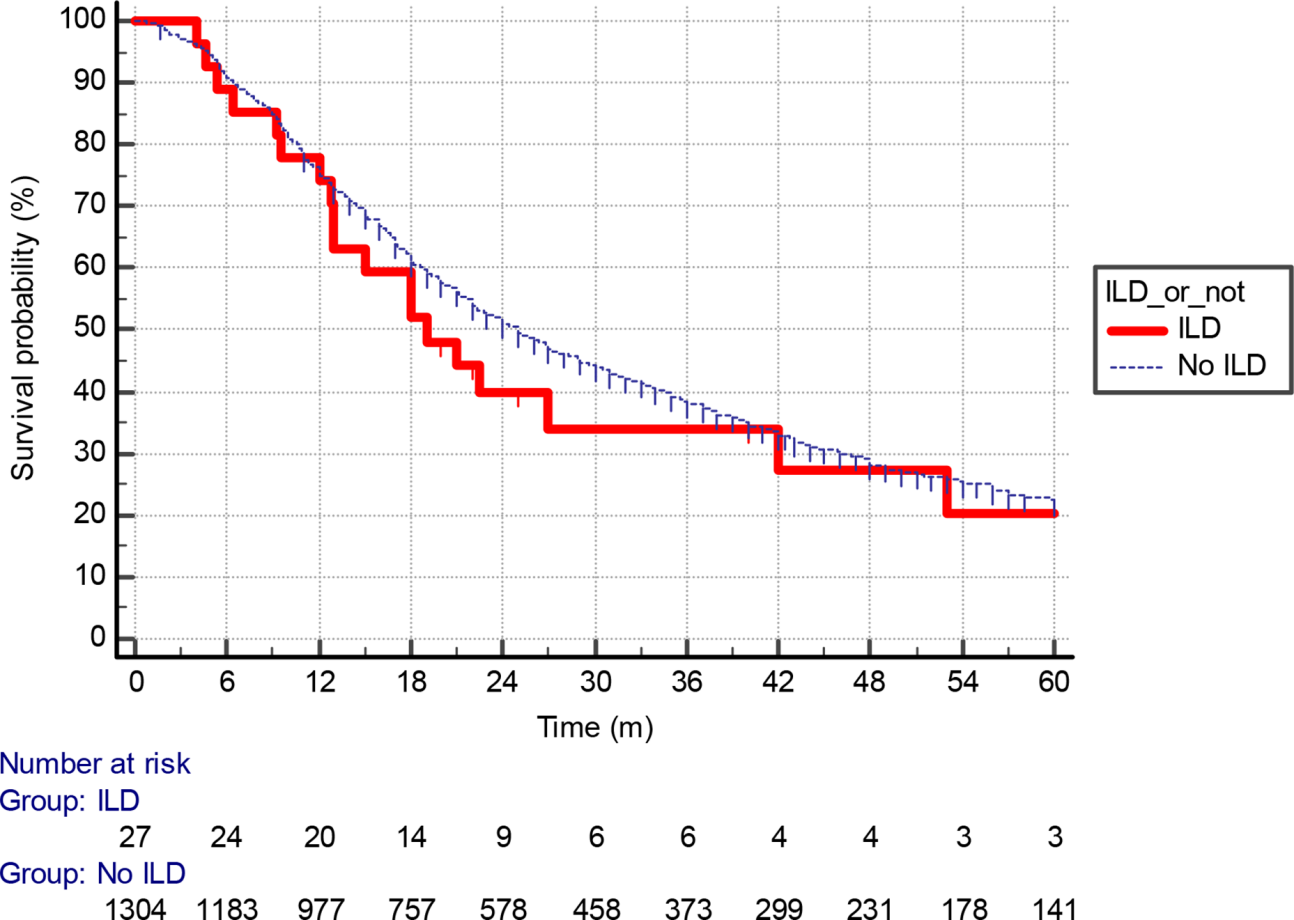
A Kaplan–Meier analysis of overall survival (months) for patients receiving radical radiotherapy stratified bypresence or absence of ILD. ILD, interstitial lung disease.

**Figure 3.**
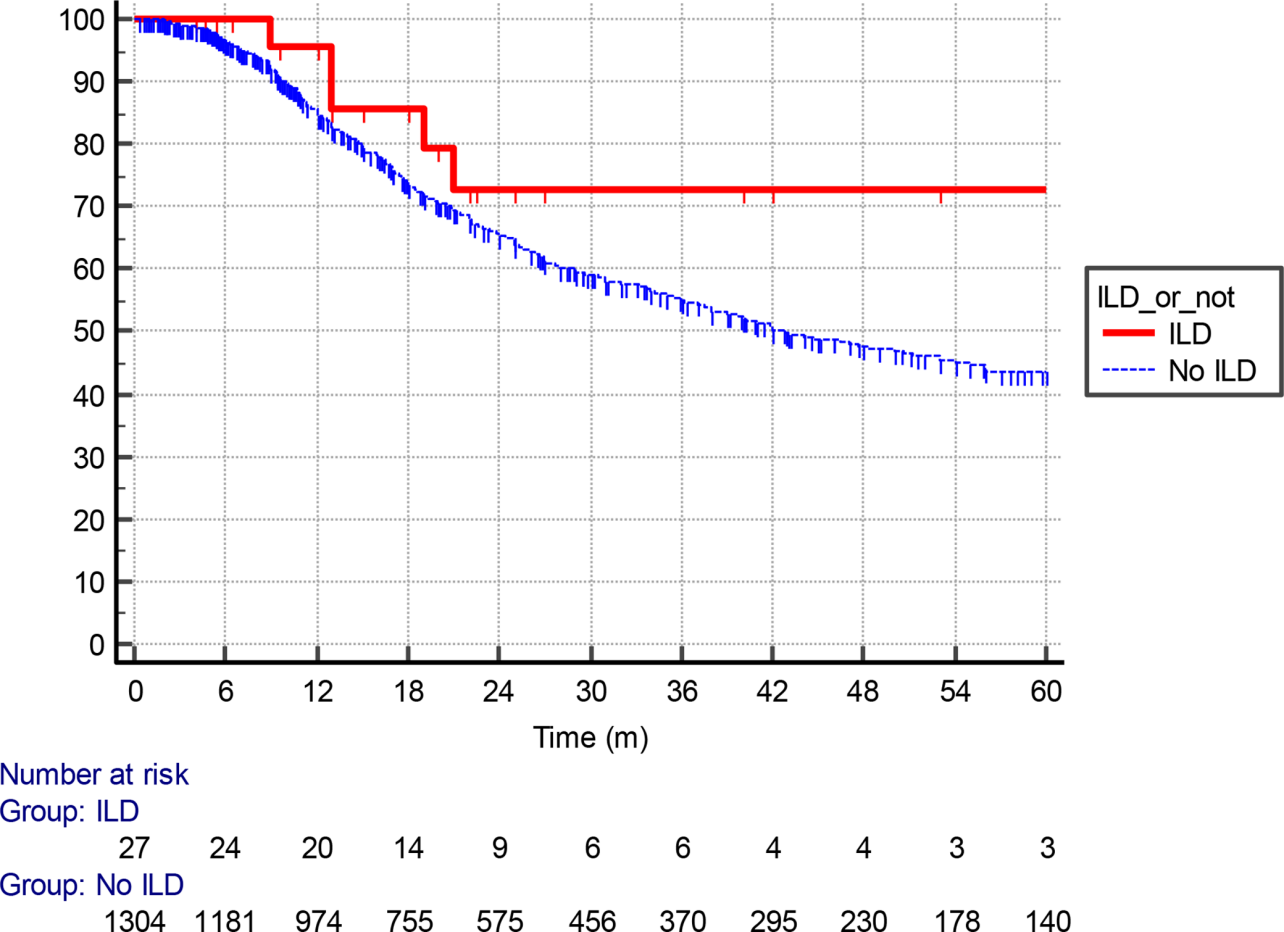
A Kaplan–Meier analysis of lung cancer-specific survival (months) for patients receiving radical radiotherapy at the Cancer Centre Belfast City Hospital stratified by presence or absence of ILD. ILD, interstitial lung disease.

**Figure 4.**
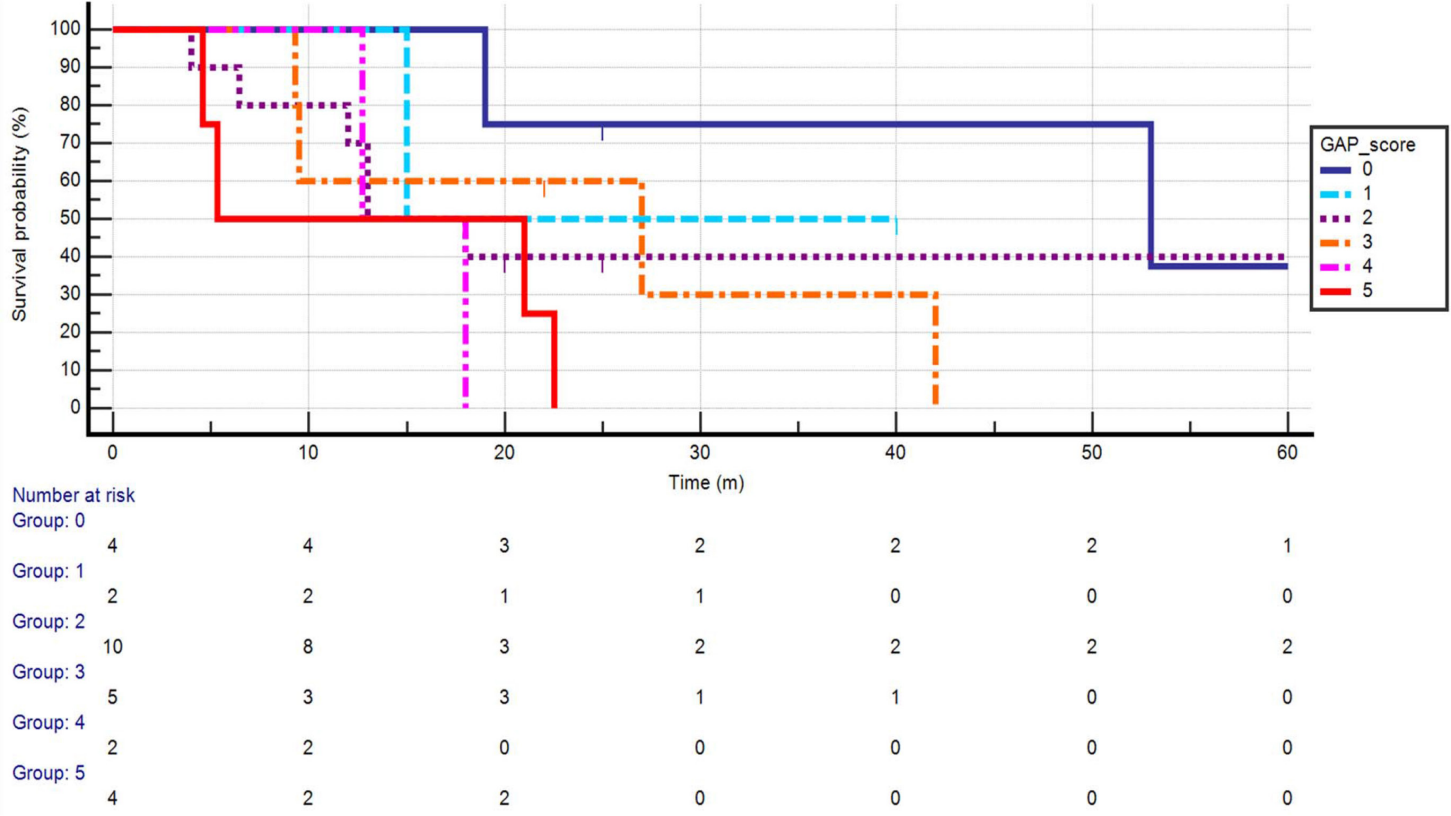
A Kaplan–Meier analysis of overall survival (months) following radical radiotherapy for lung cancer in the presence of interstitial disease, stratified by baseline ILD-GAP score. ILD, interstitial lung disease.

With a median follow-up of 13.9 months for all patients, the median OS was 19 months in the ILD cohort. This compared with 25 months in the non-ILD population (Figure 2). The HR for death was 1.14 (0.71–1.83), *p* = 0.55). Of note, the risk of death appeared elevated in the ILD cohort in the 3 years immediately following treatment. Median lung cancer specific-survival was 80 months in the ILD cohort, whereas this was 43 months (38–51) in the background cohort (Figure 3). ILD-GAP scores appropriately stratified the ILD cohort by OS duration in an exploratory analysis, as shown in Figure 4. Analysis by ILD-GAP stage was not possible as most patients were Stage I.

## Discussion

ILD describes a collective of over 150 heterogenous restrictive lung diseases characterised by non-neoplastic, non-infectious, diffuse inflammatory or fibrotic pathological features which affect the lung parenchyma.^8^ ILD is a long-established risk factor for RP following conventional radical thoracic treatment^25,26^ and the rate of ILD-specific toxicity and RT-related mortality for patients receiving SABR, were 25 and 15% respectively in a recent meta-analysis.^8^

Two-thirds of ILD cases are idiopathic with no specific recognised cause, with the remainder of cases being attributed to environmental exposures, infections, medications and collagen disorders and idiopathic pulmonary fibrosis (IPF) is the most common form.^27^ The pathophysiology for IPF is based on epithelial damage, repair abnormalities, and epithelial mesenchymal transition, whereas other subtypes have prominent features of inflammation and immunosuppression.^2,9,20^ The interaction between high-dose RT and pre-existing ILD for toxicity and survival could be explained by the role played by severalof the listed pathways in the RT pulmonary response.^28^

In the general IPF population, there is a natural incidence of acute exacerbation of 5–19% per year which often leads to respiratory failure and death leaving new lung opacities and lesions of diffuse alveolar damage.^4,8^ In patients managed surgically, patients with ILD are at higher risk of post-operative complications andrequire longer hospital admissions than those without ILD and survival is significantly lower, *i.e.* 29 *vs* 47 months.^21^

In this ‘real world’ radiotherapy study, ILD status was assessed at baseline and post-RT in terms of breathlessness, spirometry, radiological appearances and survival. The data for this prospectively evaluated cohort show that radiological deterioration of ILD following RT is almost universal, but this was not always paired with declines in pulmonary function as measured by MRCDS or spirometry. Only one-third of patients with available spirometry follow-up experienced a clinically significant fall in spirometry parameters in this small cohort. One-third of patients also went on to require home oxygen therapy. It is difficult to decipher these data however, as oxygen prescription practices vary widely, and the progressive nature of ILD necessitates supplemental oxygen prescriptions for a significant proportion of patients with ILD not treated with RT.^29,30^ One-third of patients developed grade ≥2–3 RP as a subacute radiotherapy toxicity, which is comparable to modern published series of contemporary RT planning.^31,32^ Compared to the non-ILD cohort, there was a higher rate of requiring LTOT for patients with ILD, and only ILD status and baseline FEV1 were significantly associated with requiring LTOT on multivariate analysis.

Overall survival in the presented ILD cohort was lower thanin patients without ILD treated during the same time period, although the difference was not statistically significant. Equivalent survival outcomes between ILD and non-ILD groups was also demonstrated in a published Asian cohort.^16^ The death rate in the ILD cohort observed may have been driven predominantly by the presence of ILD or other co-morbidities, as lung cancer-specific mortality appeared to be reduced in the ILD cohort. Given the competing 5-year survival rates of ILD alone, and lung cancer treated with radical RT, of 50 and 15% respectively, it is crucial that patients are given the information, support and time required to consider their priorities.^33^

At present, there are no published RT-specific tools for guiding Clinical/Radiation Oncology treatment decisions in the context of ILD. Interestingly, a previous study suggested that the standard normal tissue complication probability models are not applicable in the context of ILD, as RT dose–volume histogram parameters did not correlate with changes in pulmonary function over time following SABR.^34^

The data presented within suggest that radical RT is feasible in selected cases of mild-moderate ILD, as classified radiologically and by ILD-GAP stage. Although most patients in this cohort were WHO-PS 0–1 and did not receive chemotherapy, the average age and co-morbidity status of the cohort is reflective of the general lung cancer populationand at the Cancer Centre Belfast City Hospital, and as previously published by other centres.^35^ The dose fractionation was not altered for the majority of patients and radiation pneumonitis rates did not appear to be elevated.

The presented results suggest that ILD-GAP score may be applicable in the lung radiotherapy population. The ILD-GAP score, developed by Ley and colleagues based on a large multicentre data set, stratifies patients into three ‘stages’ of ILD based on expected prognosis and was associated with a C-index of 0.72.^36^ Subsequently, the tool incorporated ILD subtype, attributing a score of −2 for the better phenotypes, *i.e.* hypersensitivity and connective tissue disease-related ILD.^18^ Prospective validation of this tool in the setting of lung cancer RT has not been undertaken. Although too small to comprise validation, in our study a number of patients required commencement of long-term oxygen therapy, and this occurred mostly inpatient with higher ILD-GAP scores.

**Figure 5.**
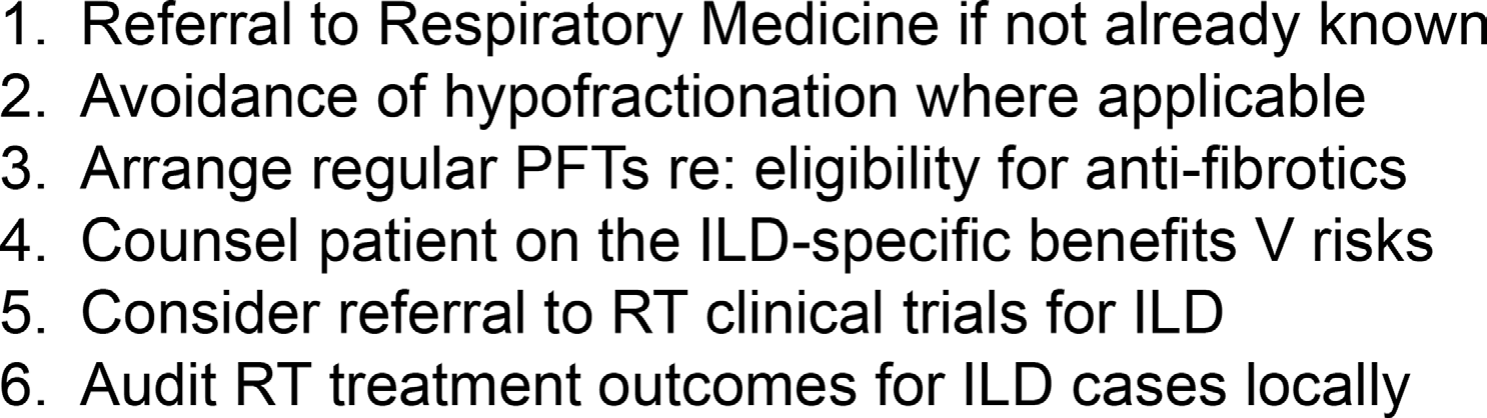
Considerations when managing radiotherapy in the case of co-occurring ILD. ILD, interstitial lung disease; RT, radiotherapy.

As well as cautious case selection for fitter patients, it is likely that improvements in RT technology, medical management and patient monitoring in recent years have contributed to the encouraging findings within our study.^37^ Most patients in this cohort received the current gold-standard for RT in terms of disease staging, motion management and planning algorithms. Furthermore, all patients were discussed at a regional thoracic oncology MDM.^38^ Alternative dose fractionations were used in some cases, such as 50Gy/5# (early disease) and 60Gy/30# (locally advanced disease), as they potentially carry a lower risk of pulmonary toxicity.^39^ Nevertheless, Clinical/Radiation Oncologists are careful to communicate the potential excess risk to survival to patients. In our department, a simple guideline document for other important considerations in this cohort was agreed by consensus amongst the thoracic oncology MDM (Figure 5).

This study had a number of weaknesses. Primarily, although the study is of a similar size to those in the recent literature,^9,15,16,40^ the findings should be interpreted with caution given the limited number of patients. Outcome data on patients referred but declined for RT were not available, so there was no directly comparable ILD cohort not receiving thoracic RT for comparison of oxygen prescriptions or survival. Hospital admission and patient-reported outcome data were not collected and no patients were commenced on antifibrotic therapy recommended by the National Institute for Clinical Excellence. Furthermore, the disease staging, dose-fractionation and respiratory follow-up intervals were not standardised. Lastly, as this was a cohort study, it is only possible to deduce possible associations, rather than conclude causative relationships exists between the variables and outcomes analysed. Taken together with other recently published series in this area, this study will inform the study design and sample size calculation for future prospective studiesand registries.

The first interventional research in the early disease setting is the recently commenced multicentre Phase II study named ASPIRE-ILD.^41^ This international study seeks to test the safety and efficacy of 50 Gy/5# in patients with ILD-GAP Score-stratified ILD, with options for dose de-escalation in the event of toxicity events. The RT offered will include motion management as standard, and dose constraints for the lung include maximum (*e.g.* V20 <10%) and minimum (*e.g.* volume of lung receiving <12.5 Gy must be >1.5 L) thresholds. Embedded translational research in a substudy will evaluate the use of MRI planning in this patient cohort. ASPIRE-ILD may provide a template for the standardised assessment of patients in the future, *e.g.* high-resolution CT monitoring, robust patient-reported outcome data collection, and regular spirometry.^41^

Challenges to be overcome in conducting high quality research in this area include the lack of a core outcome data set for reporting ILD changes on cross-sectional imaging. Distribution and severity of interstitial changes should be quantified, as well as an estimate of the overall predominant pattern.^42^ Such estimates can be difficult to make given the three-dimensional nature of the lung volumes, however, and computer-driven methods are in development, which may reduce interobserver variability.^19^ Furthermore, radiomic analyses may assist in identifying high-risk patients prospectively in the future.^43^

RT treatment decisions are complex in the population with ILD due to the additional toxicity risk introduced by this common co-morbidity and it has been shown that altered dose constraints are justifiable in SABR cases with ILD.^9^ In this study, patients with mild-moderate ILD were shown to have radiological interstitial disease progression after radical RT and borderline poorer survival compared with patients without ILD. Radiological appearances were not accompanied by spirometry and MRCDS changes in two-thirds of patients, and therefore may pose to be poor biomarkers for RT toxicity in a patient with ILD post-RT.

## Conclusions

Proceeding with radical RT in patients with confirmed mild-moderate ILD is feasible, but is associated with radiological progression of ILD and possibly reduced survival. A matching significant decline in respiratory symptoms or function after treatment observed infrequently following radical RT. For unresectable lung cancer, patients with ILD should be appropriately counselled for the additional risk of RT toxicity and clinical respiratory follow-up is advisable.

## References

[1] Kawasaki H, Nagai K, Yokose T, Yoshida J, Nishimura M, Takahashi K, et al . Clinicopathological characteristics of surgically resected lung cancer associated with idiopathic pulmonary fibrosis. J Surg Oncol 2001; 76: 53–57. doi: 10.1002/1096-9098(200101)76:1<53::aid-jso1009>3.0.co;2-t 11223825

[2] Naccache J-M, Gibiot Q, Monnet I, Antoine M, Wislez M, Chouaid C, et al . Lung cancer and interstitial lung disease: a literature review. J Thorac Dis 2018; 10: 3829–44. doi: 10.21037/jtd.2018.05.75 30069384 PMC6051867

[3] Khan KA, Kennedy MP, Moore E, Crush L, Prendeville S, Maher MM, et al . Radiological characteristics, histological features and clinical outcomes of lung cancer patients with coexistent idiopathic pulmonary fibrosis. Lung 2015; 193: 71–77. doi: 10.1007/s00408-014-9664-8 25381634

[4] Yamaguchi S, Ohguri T, Matsuki Y, Yahara K, Oki H, Imada H, et al . Radiotherapy for thoracic tumors: association between subclinical interstitial lung disease and fatal radiation pneumonitis. Int J Clin Oncol 2015; 20: 45–52. doi: 10.1007/s10147-014-0679-1 24610080

[5] Ballester B, Milara J, Cortijo J . Idiopathic pulmonary fibrosis and lung cancer: mechanisms and molecular targets. Int J Mol Sci 2019; 20(3): 593. doi: 10.3390/ijms20030593 30704051 PMC6387034

[6] Hiley C, Salem A, Batchelor T, McDonald F, Evison M . Great debate: surgery versus stereotactic radiotherapy for early-stage non-small cell lung cancer. Thorax 2020; 75: 198–99. doi: 10.1136/thoraxjnl-2019-214014 31964695

[7] Storey CL, Hanna GG, Greystoke A, AstraZeneca UK Limited . Practical implications to contemplate when considering radical therapy for stage III non-small-cell lung cancer. Br J Cancer 2020; 123: 28–35. doi: 10.1038/s41416-020-01072-4 33293673 PMC7735214

[8] Chen H, Senan S, Nossent EJ, Boldt RG, Warner A, Palma DA, et al . Treatment-Related toxicity in patients with early-stage non-small cell lung cancer and coexisting interstitial lung disease: a systematic review. International Journal of Radiation Oncology*Biology*Physics 2017; 98: 622–31. doi: 10.1016/j.ijrobp.2017.03.010 28581404

[9] Finazzi T, Ronden-Kianoush MI, Spoelstra FOB, Nossent EJ, Nijman SFM, Bahce I, et al . Stereotactic ablative radiotherapy in patients with early-stage non-small cell lung cancer and co-existing interstitial lung disease. Acta Oncologica 2020; 59: 569–73. doi: 10.1080/0284186X.2020.1730002 32079446

[10] Bahig H, Filion E, Vu T, Chalaoui J, Lambert L, Roberge D, et al . Severe radiation pneumonitis after lung stereotactic ablative radiation therapy in patients with interstitial lung disease. Pract Radiat Oncol 2016; 6: 367–74. doi: 10.1016/j.prro.2016.01.009 27068780

[11] Ueki N, Matsuo Y, Togashi Y, Kubo T, Shibuya K, Iizuka Y, et al . Impact of pretreatment interstitial lung disease on radiation pneumonitis and survival after stereotactic body radiation therapy for lung cancer. Journal of Thoracic Oncology 2015; 10: 116–25. doi: 10.1097/JTO.0000000000000359 25376512

[12] Kreuter M, Ehlers-Tenenbaum S, Schaaf M, Oltmanns U, Palmowski K, Hoffmann H, et al . Treatment and outcome of lung cancer in idiopathic interstitial pneumonias. Sarcoidosis Vasc Diffus Lung Dis 2014; 31: 266–74. 25591137

[13] Ozawa Y, Abe T, Omae M, Matsui T, Kato M, Hasegawa H, et al . Impact of preexisting interstitial lung disease on acute, extensive radiation pneumonitis: retrospective analysis of patients with lung cancer. PLoS ONE 2015; 10: e0140437. doi: 10.1371/journal.pone.0140437 26460792 PMC4603947

[14] Glick D, Lyen S, Kandel S, Shapera S, Le LW, Lindsay P, et al . Impact of pretreatment interstitial lung disease on radiation pneumonitis and survival in patients treated with lung stereotactic body radiation therapy (SBRT). Clinical Lung Cancer 2018; 19: e219–26. doi: 10.1016/j.cllc.2017.06.021 29066051

[15] Higo H, Kubo T, Makimoto S, Makimoto G, Ihara H, Masaoka Y, et al . Chemoradiotherapy for locally advanced lung cancer patients with interstitial lung abnormalities. Jpn J Clin Oncol 2019; 49: 458–64. doi: 10.1093/jjco/hyz016 30793176

[16] Kobayashi H, Naito T, Omae K, Omori S, Nakashima K, Wakuda K, et al . Impact of interstitial lung disease classification on the development of acute exacerbation of interstitial lung disease and prognosis in patients with stage iii non-small-cell lung cancer and interstitial lung disease treated with chemoradiotherapy. J Cancer 2018; 9: 2054–60. doi: 10.7150/jca.24936 29896291 PMC5995939

[17] Medical Research Council Lung Cancer Working Party*, Bleehen N, Girling D, Machin D, Stephens R . A medical Research Council (MRC) randomised trial of palliative radiotherapy with two fractions or a single fraction in patients with inoperable non-small-cell lung cancer (NSCLC) and poor performance status. Br J Cancer 1992; 65: 934–41. doi: 10.1038/bjc.1992.196 1377484 PMC1977779

[18] Ryerson CJ, Vittinghoff E, Ley B, Lee JS, Mooney JJ, Jones KD, et al . Predicting survival across chronic interstitial lung disease. Chest 2014; 145: 723–28. doi: 10.1378/chest.13-1474 24114524

[19] Robbie H, Daccord C, Chua F, Devaraj A . Evaluating disease severity in idiopathic pulmonary fibrosis. Eur Respir Rev 2017; 26: 1–12: 170051. doi: 10.1183/16000617.0051-2017 PMC9488723 28877976

[20] Raghu G, Collard HR, Egan JJ, Martinez FJ, Behr J, Brown KK, et al . An official ATS/ERS/JRS/ALAT statement: idiopathic pulmonary fibrosis: evidence-based guidelines for diagnosis and management. Am J Respir Crit Care Med 2011; 183: 788–824. doi: 10.1164/rccm.2009-040GL 21471066 PMC5450933

[21] Voltolini L, Bongiolatti S, Luzzi L, Bargagli E, Fossi A, Ghiribelli C, et al . Impact of interstitial lung disease on short-term and long-term survival of patients undergoing surgery for non-small-cell lung cancer: analysis of risk factors. European Journal of Cardio-Thoracic Surgery 2013; 43: e17–23. doi: 10.1093/ejcts/ezs560 23129356

[22] National Cancer Institute . Common Terminology Criteria for Adverse Events (CTCAE): Version 5. 2017. Available from: https://evs.nci.nih.gov/ftp1/CTCAE/About.html

[23] Pellegrino R, Viegi G, Brusasco V, Crapo RO, Burgos F, Casaburi R, et al . Interpretative strategies for lung function tests. Eur Respir J 2005; 26: 948–68. doi: 10.1183/09031936.05.00035205 16264058

[24] Zappala CJ, Latsi PI, Nicholson AG, Colby TV, Cramer D, Renzoni EA, et al . Marginal decline in forced vital capacity is associated with a poor outcome in idiopathic pulmonary fibrosis. Eur Respir J 2010; 35: 830–36. doi: 10.1183/09031936.00155108 19840957

[25] Makimoto T, Tsuchiya S, Hayakawa K, Saitoh R, Mori M . Risk factors for severe radiation pneumonitis in lung cancer. Jpn J Clin Oncol 1999; 29: 192–97. doi: 10.1093/jjco/29.4.192 10340042

[26] Sanuki N, Ono A, Komatsu E, Kamei N, Akamine S, Yamazaki T, et al . Association of computed tomography-detected pulmonary interstitial changes with severe radiation pneumonitis for patients treated with thoracic radiotherapy. J Radiat Res 2012; 53: 110–16. doi: 10.1269/jrr.110142 22302051

[27] Kanaji N, Tadokoro A, Kita N, Murota M, Ishii T, Takagi T, et al . Impact of idiopathic pulmonary fibrosis on advanced non-small cell lung cancer survival. J Cancer Res Clin Oncol 2016; 142: 1855–65. doi: 10.1007/s00432-016-2199-z 27350261 PMC4954838

[28] Käsmann L, Dietrich A, Staab-Weijnitz CA, Manapov F, Behr J, Rimner A, et al . Radiation-Induced lung toxicity – cellular and molecular mechanisms of pathogenesis, management, and literature review. Radiat Oncol 2020; 15: 1–16. doi: 10.1186/s13014-020-01654-9 PMC7488099 32912295

[29] Bell EC, Cox NS, Goh N, Glaspole I, Westall GP, Watson A, et al . Oxygen therapy for interstitial lung disease: a systematic review. Eur Respir Rev 2017; 26: 160080. doi: 10.1183/16000617.0080-2016 28223395 PMC9489021

[30] Hardinge M, Annandale J, Bourne S, Cooper B, Evans A, Freeman D, et al . BTS guidelines for home oxygen use in adults. Thorax 2015; 70: i1–43. 25870317 10.1136/thoraxjnl-2015-206865

[31] Zhang T, Zhou Z, Bi N, Wang J, Wang L, Deng L, et al . Vmat for unresectable locally advanced NSCLC does not increase the risk of radiation pneumonitis compared with IMRT. International Journal of Radiation Oncology*Biology*Physics 2019; 105: E543. doi: 10.1016/j.ijrobp.2019.06.2464

[32] Wu K, Xu X, Li X, Wang J, Zhu L, Chen X, et al . Radiation pneumonitis in lung cancer treated with volumetric modulated Arc therapy. J Thorac Dis 2018; 10: 6531–39. doi: 10.21037/jtd.2018.11.132 30746197 PMC6344715

[33] Koo S-M, Uh S-T, Kim DS, Kim YW, Chung MP, Park CS, et al . Relationship between survival and age in patients with idiopathic pulmonary fibrosis. J Thorac Dis 2016; 8: 3255–64. doi: 10.21037/jtd.2016.11.40 28066605 PMC5179452

[34] Guckenberger M, Klement RJ, Kestin LL, Hope AJ, Belderbos J, Werner-Wasik M, et al . Lack of a dose-effect relationship for pulmonary function changes after stereotactic body radiation therapy for early-stage non-small cell lung cancer. International Journal of Radiation Oncology*Biology*Physics 2013; 85: 1074–81. doi: 10.1016/j.ijrobp.2012.09.016 23154077

[35] Sun F, Franks K, Murray L, Lilley J, Wheller B, Banfill K, et al . Cardiovascular mortality and morbidity following radical radiotherapy for lung cancer: is cardiovascular death under-reported? Lung Cancer 2020; 146: 1–5. doi: 10.1016/j.lungcan.2020.05.004 32460218

[36] Ley B, Ryerson CJ, Vittinghoff E, Ryu JH, Tomassetti S, Lee JS, et al . A multidimensional index and staging system for idiopathic pulmonary fibrosis. Ann Intern Med 2012; 156: 684–91. doi: 10.7326/0003-4819-156-10-201205150-00004 22586007

[37] Brown S, Banfill K, Aznar MC, Whitehurst P, Faivre Finn C . The evolving role of radiotherapy in non-small cell lung cancer. Br J Radiol 2019; 92: : 20190524. doi: 10.1259/bjr.20190524 31535580 PMC6913359

[38] Heinke MY, Vinod SK . A review on the impact of lung cancer multidisciplinary care on patient outcomes. Transl Lung Cancer Res 2020; 9: 1639–53. doi: 10.21037/tlcr.2019.11.03 32953538 PMC7481642

[39] Goodman CD, Nijman SFM, Senan S, Nossent EJ, Ryerson CJ, Dhaliwal I, et al . A primer on interstitial lung disease and thoracic radiation. J Thorac Oncol 2020; 15: 902–13. doi: 10.1016/j.jtho.2020.02.005 32105810

[40] Al Feghali KA, Wu QC, Devpura S, Liu C, Ghanem AI, Wen NW, et al . Correlation of normal lung density changes with dose after stereotactic body radiotherapy (SBRT) for early stage lung cancer. Clin Transl Radiat Oncol 2020; 22: 1–8. doi: 10.1016/j.ctro.2020.02.004 32140574 PMC7047141

[41] Palma DA, Chen H, Bahig H, Gaede S, Harrow S, Laba JM, et al . Assessment of precision irradiation in early non-small cell lung cancer and interstitial lung disease (ASPIRE-ILD): study protocol for a phase II trial. BMC Cancer 2019; 19: : 1206. doi: 10.1186/s12885-019-6392-8 31829203 PMC6905060

[42] Romei C, Tavanti L, Sbragia P, De Liperi A, Carrozzi L, Aquilini F, et al . Idiopathic interstitial pneumonias: do HRCT criteria established by ATS/ERS/JRS/ALAT in 2011 predict disease progression and prognosis? Radiol Med 2015; 120: 930–40. doi: 10.1007/s11547-015-0526-0 25743239

[43] Bernchou U, Hansen O, Schytte T, Bertelsen A, Hope A, Moseley D, et al . Prediction of lung density changes after radiotherapy by cone beam computed tomography response markers and pre-treatment factors for non-small cell lung cancer patients. Radiother Oncol 2015; 117: 17–22. doi: 10.1016/j.radonc.2015.07.021 26255762

